# Collaboration with general practitioners: preferences of medical specialists – a qualitative study

**DOI:** 10.1186/1472-6963-6-155

**Published:** 2006-12-04

**Authors:** Annette J Berendsen, Wim HGM Benneker, Jan Schuling, Nienke Rijkers-Koorn, Joris PJ Slaets, Betty Meyboom-de Jong

**Affiliations:** 1 Department of General Practice, University Medical Centre Groningen, University of Groningen, Antonius Deusinglaan 1, 9713 AV Groningen, The Netherlands; 2 Department of Internal Medicine, University Medical Centre Groningen, University of Groningen, The Netherlands

## Abstract

**Background:**

Collaboration between general practitioners (GPs) and specialists has been the focus of many collaborative care projects during the past decade. Unfortunately, quite a number of these projects failed. This raises the question of what motivates medical specialists to initiate and continue participating with GPs in new collaborative care models.

The following question is addressed in this study: *What motivates medical specialists to initiate and sustain new models for collaborating with GPs?*

**Methods:**

We conducted semi-structured interviews with eighteen medical specialists in the province of Groningen, in the North of The Netherlands. The sampling criteria were age, gender, type of hospital in which they were practicing, and specialty. The interviews were recorded, fully transcribed, and analysed by three researchers working independently. The resulting motivational factors were grouped into categories.

**Results:**

'Teaching GPs' and 'regulating patient flow' (referrals) appeared to dominate when the motivational factors were considered. In addition, specialists want to develop relationships with the GPs on a more personal level. Most specialists believe that there is not much they can learn from GPs. 'Lack of time', 'no financial compensation', and 'no support from colleagues' were considered to be the main concerns to establishing collaborative care practices. Additionally, projects were often experienced as too complex and time consuming whereas guidelines were experienced as too restrictive.

**Conclusion:**

Specialists are particularly interested in collaborating because the GP is the gatekeeper for access to secondary health care resources. Specialists feel that they are able to teach the GPs something, but they do not feel that they have anything to learn from the GPs. With respect to professional expertise, therefore, specialists do not consider GPs as equals. Once personal relationships with the GPs have been established, an informal network with incidental professional contact seems to be sufficient to satisfy the collaborative needs of the specialist.

The concerns seem to outweigh any positive motivational forces to developing new models of collaborative practice.

## Background

The collaboration between specialists and general practitioners (GPs) has important implications for health care systems where the GP controls patient access to specialist care.

New models of collaboration between specialists and GPs are assumed to improve the efficiency of patient care and to contribute to decreasing costs particularly in cases of chronic illness [1].

Specialists are professionals in the same sense that GPs are. Professionalism is characterized by clearly demarcated work and knowledge domains, special training, and assessment [2,3]. Professional autonomy, which also includes participating in management or medical leadership, is an important motivating factor for professionals. It is therefore important, when developing new models of collaboration, to take into account the interests and the needs of the professionals.

During the past decade, in Great Britain and in The Netherlands, experience has been gained in the development and organization of new collaboration models. The barriers to collaboration are almost the same in these two countries [4]. They include structure, procedures, finance and legitimacy at system and institutional level as well as the professional self-interest at the operational level. The professional barriers were: -competing ideologies and values; -professional self-interest and autonomy, and inter-professional competition for domains; -conflicting views about patients' interests and roles.

Changes are necessary in the manner in which physicians carry out their professional duties and how they perceive their role in the medical profession [5,6].

During the late nineteen-nineties, *qualitative *methods were used to examine the opinions of specialists concerning working collaboratively with GPs [7-9]. However, although considerable attention was paid to the relationship between the specialist and the GP, almost no attention was given to new forms of working collaboratively. Other *qualitative *research focused on special topics such as the prescribing of specialist medication [10], collaborative care for patients with rheumatism [11], and the concept of 'hospital at home' [12]. The remaining literature was *quantitative *in nature [13-16].

The implementation of changes seems to depend for an important part on professionals working collaboratively. This collaboration is necessary both to develop and initiate new forms of collaboration and to implement them in the regular care setting. In another study we examined the opinions and preferences of GPs [17]. In the present study, we conducted a qualitative analysis examining the views of the specialist. Using an interview format, we asked them why they would want to work collaboratively, which factors motivated them to work collaboratively, and whether these factors would endure.

This led to the following research question:

What motivates specialists to initiate new forms of working collaboratively with GPs and will these motivational forces last?

No research addressing this question was found in the literature.

## Methods

We chose an exploratory qualitative research design, as little is known about the preferences of specialists with respect to the development of new collaborative practice models. We defined 'new collaborative practice models' as types of contact about a patient other than the conventional contact through correspondence or telephone. As personal motives play an important role in this area, we chose a format consisting of semi-structured interviews to gain as many personal insights as possible. Ethical approval was not required.

### Research population

We selected a purposive sample of nineteen specialists in order to obtain different opinions and experiences. The research was conducted in the Province of Groningen in the North of The Netherlands. Sampling was done on gender, age, specialty, and whether or not the physician worked in an academic centre, a highly rated clinical hospital, or a peripheral hospital. We choose medical specialists known by GPs for their critical attitude towards collaboration as well as medical specialists known by GPs for their collaborative attitude. The specialists were divided into three categories according to their specialty: twelve physicians (which included internists, paediatricians, and psychiatrists), five surgeons (which included gynaecologists), and two supporting specialists (which included radiologists). Only specialists involved directly in patient care were included.

### Data gathering

The interviews were conducted by two GPs, each trained and experienced in conducting interviews and familiar with exploratory interview methods. In earlier research, it was reported that the advantages of a medical colleague conducting the interview outweighed the disadvantages [7]. These advantages include a simplified introduction, an improved understanding, and the opportunity to challenge the interviewees. The goal of the research was explained prior to each interview. Subjects were also told that anonymity would be preserved during data analysis. The subjects were encouraged not to concern themselves about expressing criticism about GPs even though the interviewer was a GP.

The interviews were semi-structured and focused on the professional role of the specialist. The topics for discussion were: positive and negative experiences with GPs and with new forms of collaboration; and personal objections and preferences when working collaboratively. The subjects were asked to use concrete examples to illustrate their opinions. The questions did not have to follow a specific order to allow the subjects to freely associate among different topical areas. This caused some topics to be discussed in depth.

### Analysis

Each interview was recorded on audiotape and later transcribed verbatim. Working independently, three researchers (two GPs and one medical student) assigned labels to what they felt to be the most important statements in the complete transcripts. Implicit as well as explicit statements were analyzed. The researchers then discussed any discrepancies in the findings until a consensus was reached. The transcripts were also read by a senior researcher to control for short, open and neutral questions. The analysis was conducted according to the methods of "Qualitative Data Analysis Methodology" (QDA) and the "framework" method based on elements from "grounded theory" [18,19]. The five most important steps were: familiarization, identifying a thematic framework, indexing, charting, and mapping/interpretation. The actual interviews and the analyses were conducted almost simultaneously, so that the researchers could control for topic saturation. For the analysis, the computer program Kwalitan 5.0 was used. This allowed for the identification of relevant themes. The identified motivational themes were ranked.

## Results

Of the 19 specialists approached, 18 agreed to participate in the research. One surgeon from a peripheral hospital did not wish to participate due to time constraints. The ratio of men to women was 14:4, and the ages ranged from 36 to 57 years. Ten of the specialists were working in a university hospital setting, six in a leading general hospital, and two were working in a peripheral setting. The interviews each lasted approximately one hour, and generally took place in the hospital setting. The interviews were conducted between April 2004 and January 2005 inclusive.

Ranking the themes took place after four interviews and again after fifteen interviews were completed. From the sixteenth interview onward, no new views were being brought forward; saturation had been reached. In view of the purposive sampling all eighteen interviews were nevertheless conducted.

**Figure 1:**
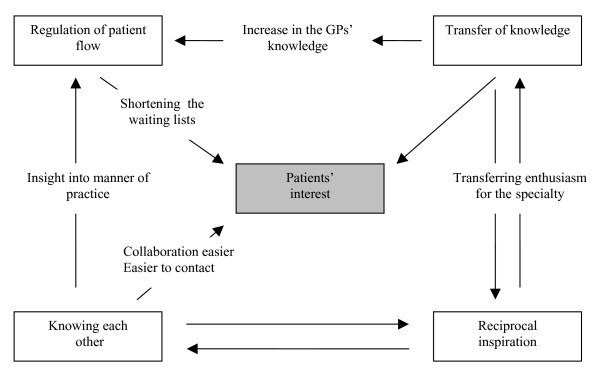
The most important motives for specialists to work collaboratively.

Figure 1 shows the most important motives to work collaboratively with their underlying relationships.

### Patients' interest

All of the specialists interviewed felt that the primary goal of developing new collaborative practices should be an improvement in patient care. A number of specialists said that through collaboration, one can deliver improved care, thereby improving the quality of life of the patient.

A number of subjects tended to favour an optimal type of incidental collaboration rather than structured collaboration.

On the other hand, other specialists favoured a multi-disciplinary (multi-specialty) outpatient clinic as this would be in the best interests of the patient.

The specialists felt that patients should be cared for by a qualified team, possibly with the GP in a central role. For this to work effectively, however, everybody involved has to have a good understanding of the common goals. Some subjects felt that this requirement might present problems. Multidisciplinary clinics were proposed, including geriatrics, palliative care, oncology, dermatological nursing for chronic wounds, and comprehensive diagnostics. The GP would be able to contribute by assessing possible complications of a treatment with his background knowledge of the patient and his or her social and family circumstances.

Some did see the advantage of following a common set of guidelines. This applied exclusively to agreements concerning how the referral process should take place. Comprehensive diagnostic, for example, could follow guidelines according to which the problems of the patient are assessed and addressed in a single day eliminating the need for a multitude of visits. This would be convenient for the patient. Work within protocols could be done, for example, by nurse practitioners.

Some specialists initially expressed support for the idea of combined office hours, but added that the time investment would be prohibitive and besides, they already have enough work.

*Yeah, the sicker the patient, the more important it is to, these people don't have a lot of time. You have to be able to use patients' time efficiently*.

### Regulating patient flow

Nearly all of the specialists felt that solving problems in the primary health care setting saves time by eliminating the need for a consult. This would apply, for example, to back, neck, and shoulder complaints, dizziness, and headache. This can improve wait times and reduce waiting lists, and that is in the best interests of the patient. The respondents indicated that this should be promoted, because the specialists already have enough work. The pressure on secondary health care providers could be decreased if GPs were to do some of the necessary check-ups themselves. Much care can be transferred to the GPs and this has to be well coordinated.

*Projects are initiated, not so much because the GP will like it, but we are simply getting too busy*.

*Yes, there are of course many colleagues in peripheral areas, who will blame me if I say this. They then say that that is our "core business", but I think that this should be able to be done more efficiently and more cheaply in and around the GPs' practices*.

*Good collaboration takes the pressure off of secondary health care, and that's what we want. It is good for the patient and therefore also good for the national economy, because you end up solving a number of problems in the primary health care setting*.

### Transfer of knowledge

#### Increase in the knowledge of the GP

Almost all specialists find time spent teaching to be time well spent. They enjoy teaching the GPs and making them enthusiastic for the profession. The specialists would like to give the GPs the necessary tools, so that they can handle certain problems and so that when they do refer a patient, the patient will be better prepared. If the specialists are involved in the continuing education of GPs, they would also be better able to determine the level of the GPs' knowledge.

*And when the GP loses his way doing the physical assessment or taking case history from the patient, look, it is very nice to, smart aleck that I am..., to point out little tricks of the trade*.

The specialists who were interviewed think that contributing to the medical knowledge of GPs is an ongoing process. They felt this to be a good argument for the development of new forms of collaboration. The respondents also felt that the GPs should be kept informed of the new developments in their field. A few of the specialists stated that intensive additional education was especially useful for the GP who wants to specialize in a certain area. This GP with a Special Interest would then be able to guide and coordinate the continuing education of a large group of GPs. Moreover, this GP would be able to fulfil a bridging function in collaborative care settings.

#### Increase in the knowledge of the specialist

Almost none of the specialists thought they could learn anything from the GPs. The occasional respondent thought they could gain insights into how a GP handles uncertainties.

Some specialists said that they could respect the primary position of the GP and his or her function as gatekeeper to the other levels of health care. They therefore found it important to understand how the GP categorizes patients.

### The personal relationship between the GP and the specialist

#### Knowing each other personally: collaborating more easily

Many specialists find it important to know the GPs personally. They find that their work becomes easier and more pleasurable as a result. When people know one another personally, it is easier to reach each other for consultation and this is to the benefit of the patient. The quality and efficiency of telephone consultations improve and the specialist gains added insight into how the GP functions.

*I think that true collaboration is not possible in medicine, unless you know each other better, more personally. That is not simply achieved through a change in format*.

*When a GP comes on the line whom I've spoken to there, then I think, oh yeah, that's that GP. Then, well, that just makes the contact easier*.

#### Insight into manner of working

When one doesn't know the GP, the specialists stated that it is difficult to gauge the knowledge of the GP. They also said that over the course of time, they are gradually able to form a picture of someone's manner of working.

All the specialists indicated that there are differences in interest and quality among GPs. They found some to be enthusiastic and quick to take over patient care when necessary. Other GPs, however, were seen as being very tiresome.

*I know the names. I see a big problem developing with a patient and then I look at the ID tag and then I think: oh, this guy's in good hands*.

*If someone, Dr. X yells "wolf" then I know something's up. When Dr. Y yells "wolf" then I think to myself: okay, no rush, it's Dr. Y the alarmist. And that's how it goes*.

*That some GPs use a big whip to take care of their patients with problems by referring them in all directions. That also gives us a certain picture. Those are also *not *the people who will be calling us*.

### Reciprocal inspiration

A number of specialists said that they enjoy working collaboratively with GPs to find solutions for identified problems.

*If I, with GPs, am able to solve a complex problem, when I can contribute something, when I have the feeling that my expertise and my experience were necessary to do that, then, well, that gives me a real thrill, that gives me a very good feeling. And that is a thrill that, that you don't experience the same way if you are only dealing with patients and are not working together with your colleagues*.

### Other aspects of collaboration

#### Status

Most specialists said that they did not experience a difference in status, because they are not sensitive to it. Some did say, however, that they had observed an arrogant attitude among their colleagues toward GPs.

A few of the respondents stated that there is a smaller difference in status now than there has been historically, partially because specialty training focuses increasingly on working in a team setting.

A number of specialists said that they notice from the GPs' attitudes, that the GPs regard the specialists as having a higher status. They concluded this from the way the GP addressed the specialist and from a certain amount of defensiveness in their behaviour. The specialists said that they did not like that.

*Well, I have always had the idea that people think that a specialist has a higher status than a GP, that he should have a higher status. But I don't see it that way*.

*I feel it, but I don't get attached to the feeling. Of course, it strokes your ego when you are treated as though you have a high status; on the other hand, this is not what I am after*.

*There is nothing worse than ruining a potentially beneficial collaboration by having the wrong attitude..., but you do hear, from time to time, that it's a problem, that some specialists place themselves on a sort of pedestal anyway*.

*The GP runs his practice. We run, in fact, an ****entire ****clinic*.

Some of the specialists stated that they have noticed a difference in status among specialists. For example, they ascribed a lower status to dermatologists and psychiatrists. Some of these specialists in turn indicated that they do indeed have to work harder at being taken seriously.

A number of specialists find that it is important for the day to day functioning to have a certain amount of status with respect to the patient. After all, patients should not be able to walk all over you. Specialists are of the opinion that patients grant them a higher status overall than GPs; the GP is more familiar for the patient.

Psychiatrist: *You've heard the well known joke about us: they know nothing and they do nothing*.

*I come back from Africa, one of my great aunts, she sees me at a party, walks toward me and says: 'what do I hear now? I thought you were going to become a surgeon and now you are going to become a radiologist. How AWFUL.' Well, that was actually typical*. Laughter.

*I don't need to be put on a pedestal, but medicine is a serious profession and people/patients have to be aware of that*.

##### The present relationship between the GP and the specialist

Some specialists feel that the relationship between the GP and the patient is becoming more distant. They say that this could be due to the fact that the GP is busier. Many specialists also feel that the character of primary care is changing. There are some GPs who are becoming less involved because they more often work part-time and because they follow stricter hours. The trusted physician who visits you at home after hours is gradually disappearing. This is why some specialists find it difficult to form a network with the GP around a particular family with medical problems. This general trend stands in the way of a closer collaborative relationship.

*When someone is covering for another physician, and they phone me and say: 'sorry, but I'm here with one of this guy's patients and I don't know the history, I don't know the exact diagnosis, and I don't know what medication the patient is on', that really irks me*.

*That's the part-timer. That's the GP who locks his doors at four o'clock and really has no inclination to make that extra patient visit after four*.

*There is now a new generation of doctors. And the old generation is picking up some of this as well. Especially the new generation though is inclined, these are no longer the 8 to 5 doctors, I call them the 9 to 4:30 doctors, they live and work in the city and go home like a shot when they're done. And yes, outside of office hours, they're no good to you at all*.

Some of the specialists mentioned that developments among specialists will also have a negative effect on collaborative practice. There are more and more subspecialties which address increasingly narrow areas of medicine. This requires better collaboration with GPs and also among specialists.

Cardiologist: *In a few years we'll have one specialist for the left ventricle and another for the right*.

### Resistance to change

Almost all of the specialists perceived many, often unscalable barriers to increasing or changing collaborative care practices. Table 1 shows the most important negative themes.

#### Personal investment, time and energy

A large number of specialists shared the opinion that projects can only be successful if there is a personal investment by devoted people of whom there are not enough. You have to know what you have to offer each other, you have to fulfil your commitments, endure, dare to make a beginning and, above all, not have too many meetings. The respondents said that it is better to start on a small scale, with a minimal number of intermediate levels. Adjustments can always be made later. The GP should be able to communicate directly with the specialist.

They think that it would be better to limit themselves to a number of simple interventions. Nobody is interested in projects that involve too much paperwork; besides, they are too busy for this. Projects are often too complex and the respondents stated that this leads to resistance.

*Two years of project funding had already been invested in that initiative. All that was left was empty waffling, such a thick report of what all had to happen, nothing had been done... and yeah, there's a lot of administrators on board, a lot of bla-bla, if you see the report, it's made up of evaluation, implementation, project, trajectory, it is one big bla-bla jargon. That is an entirely self-perpetuating circuit*.

Many respondents asked themselves how much energy would be necessary for a new project and what the outcome would be. They are reluctant out of a fear that new initiatives will cost a lot of time. They felt that you can not simply add collaborative practice as something 'on the side' parallel with running the day to day practice.

*If we don't look out, our entire staff will spend the entire day being multidisciplinary with each other*.

#### Foundation

Many specialists do not feel supported by their colleagues when they make changes. According to them, a project fails when people do not come through according to previous agreements. This leads to increasing irritation and the eventual cancellation of the project. Besides, some of them find it difficult to take initiative, because they are afraid that they will be left to carry the load in the end.

Some specialists want to give priority to collaboration among themselves before they focus their energies on collaborating with the GPs.

A number of specialists said that they get the impression from GPs that the GPs are not interested in working together. This happens because the GPs see few of that specialist's patients or because, for example in the palliative care setting, the care already in place is well-organized.

*If I'm away, it should, above all, not affect anyone else. So I have to do it on the side, it's tolerated*.

Because I see, if you start something, you'll never be rid of it, get it?

#### Remuneration

Many specialists say that new forms of collaborative practice are not possible because they do not profit the specialist. Office hours produce more revenue than collaborative projects even when one is on salary. In the academic setting, if one sees more patients, one can also do more research. More research means more PhD theses, more articles, more academic output, and, indirectly, more money.

One of my concerns is, if we have to do more of that sort of thing, how will we organize that and with what kind of organization?

The respondents did feel that new initiatives must be well structured and well organized. The necessary facilities, manpower, and equipment must be obtained. At the start of a project, they said, there is usually financial support, because the health care insurance companies also find it important. But, if something is initiated and is running well, then there is no additional funding to continue the project.

#### Common guidelines

Some specialists find that their profession is not one that can be rigidly demarcated and find guidelines too restrictive.

Others find that common guidelines are a good idea. They also feel that there then has to be a kind of longitudinal case manager who will keep the participants on task. They find it a disadvantage that more and more people are required who then also have to be supervised.

## Discussion

### Key findings

If new forms of collaboration between professionals are initiated, they have to dovetail with the needs and the interests that professionals have.

Specialists identified the regulation of patient flow as one of the primary aims for collaborative care. They would like to see the pressure on outpatient clinics reduced, as this is in the best interests of the patient. Moreover, specialists want to cooperate in the improvement of the quality of the referral. This is part of the reason for spending energy on giving the GPs further training. Most specialists are under the impression that GPs have little or nothing to teach them.

Specialists would also like to get to know GPs on a more personal level, because they believe that working together leads to greater job satisfaction. Although we expected that one would find it less important to get to know each other, in the present day situation with more and more part-time GPs and subspecializations, the specialists clearly identified this as a need.

The most important concerns raised by the specialists were 'lack of time', 'no collegial support', and 'no financial compensation'. Many specialists find guidelines too binding. Moreover, many projects were experienced as being too complex and time consuming.

### Study limitations and strengths

All the specialists experienced the interview as positive; it allowed them to organize their ideas about this topic. The transcripts showed that the specialists expressed themselves freely, although they tended to phrase their thoughts politely.

Our study was undertaken within one province of the Netherlands and our findings may not be wholly representative of attitudes in other areas. However, the province of Groningen offers areas with contrasting demography.

The researchers were aware and alert to the possibility of bias during the research. Everything possible was done to decrease, minimalize, or eliminate the effect of possible bias on the interviews and on the interpretations. Additionally, the results were discussed with many different people. Thus we also experienced, as reported in another study, that the advantages amply made up for the possible disadvantages [7].

The strengths of this study are the qualitative method that really captured motivational factors and it's the first of its kind in this field.

### Relating the results to the existing literature

Collaborative practice may be in the patient's best interest. Research has shown that a patient with heart failure who is followed by the GP working collaboratively with the specialist receives significantly better care [20].

Regulating patient flow is up till now not a recognized motivational factor for working collaboratively. Earlier research has shown specialists' attitude with respect to the coordinating role of the GP is influenced by their financial interest [16]. Specialists would rather have check-ups taking place in hospital when that produces income [10].

Specialists are not always satisfied with the examination done or the care delivered by the GP preceding the referral [13]. This agrees with the finding that specialists are willing to collaboratively develop guidelines for the referral process.

Earlier research showed that specialists feel they have much to teach GPs but little to learn from them [9]. Specialists were also shown to prefer concentrating on new developments in their area of specialty [8]. The specialists included in the present study also made clear mention of this. The desire to develop relationships with the GPs on a personal level appears to be consistently present [7,11].

It is known that specialists are resistant to guidelines in general. They are less inclined to accept them[14], and they are afraid that they will lose their autonomy [21]. Specialists additionally feel that guidelines cause an increase in workload, there is no adequate financial compensation, and there is a shortage of the necessary personnel [22].

## Conclusion

Specialists are particularly interested in collaborating because the GP is the gatekeeper for access to secondary health care resources. Working together to decrease wait-times and to decrease the pressure on the outpatient clinics therefore seems to be the strongest argument to change the collaborative structure of care.

Specialists consider knowledge transfer to GPs to be an ongoing process. GPs, on the other hand, would rather, at certain moments, shift their focus to another specialty. Specialists feel that they are able to teach the GPs something, but they do not feel that they have anything to learn from the GPs. With respect to professional expertise, therefore, specialists do not consider GPs as equals. This may be more than a simple difference in expertise as it may also indicate an underlying difference in status. Such a hierarchy causes asymmetry between specialists and GPs and might be an important barrier to collaborative practices.

Specialists consider it important to develop personal relationships with GPs. Initiatives with this in mind should be stimulated. However, it seems that once this fundamental relationship has been formed, an informal network is sufficient.

These motivational forces will probably not last, unless problems concerning access to specialist care persist.

Most of the specialists interviewed raised many concerns. This is probably the reason that people who wish to work together look for a solution involving a minimum of complexity. This should be taken into account when developing new initiatives.

This study leads us to conclude that for new successful collaborative practices to be developed, the cost benefit ratio must be improved, because the concerns voiced by the specialists seem to outweigh the positive motivational factors.

## Note

In another study we examined the opinions and preferences of GPs [17]. Specialists did not suggest many new collaboration models in contrary to the GPs. If specialists did so they instantly came up with barriers. That is why no new models are described in this paper and more attention is paid to specialists' barriers.

## Competing interests

The author(s) declare that they have no competing interests.

## Authors' contributions

All authors except NR-K contributed to the design of this study. AJB was responsible for the day-to-day management and produced the first draft of the manuscript. AJB and WHGMB conducted the interviews and analyzed them together with NRK and JS. All authors contributed to the write-up of this study. All authors read and approved the final manuscript.

## Pre-publication history

The pre-publication history for this paper can be accessed here:



## Figures and Tables

**Table 1:** Specialists' resistance to adopt new collaborative practice models

**Personal**	Little motivation
	Lack of time
	• too much discussion
	• too much paperwork
	• too complicated project
	
	Insufficient interest among GPs
	
**Foundation**	One stands alone
	• no support from colleagues
	• colleagues appear uninterested
	• nobody wants to take over the responsibility later
	
**Remuneration**	Time investment is not rewarded
	Lack of money for continuation of project
	
**Common guidelines**	Too restrictive
	Oversight necessary for involved physicians
